# Sézary syndrome associated vitiligo-like leukoderma in type V skin: Report of a case and review of the literature

**DOI:** 10.1016/j.jdcr.2024.04.009

**Published:** 2024-04-15

**Authors:** Fatima N. Mirza, Adam J. Olszewski, Ari R. Pelcovits, Christopher DiMarco, Elnaz F. Firoz

**Affiliations:** a Department of Dermatology, Warren Alpert Medical School of Brown University, Providence, Rhode Island; b Department of Hematology and Oncology, Warren Alpert Medical School of Brown University, Providence, Rhode Island

**Keywords:** cutaneous T-cell lymphoma, Sézary syndrome, vitiligo-like leukoderma

## Introduction

Cutaneous T-cell lymphoma (CTCL) is a collective term used to refer to a heterogeneous group of skin-limited and systemic cutaneous lymphomas of skin-homing T cells. Both neoplastic and inflammatory processes may lead to pigmentary changes which can be the hallmark of certain subtypes of CTCL. Hypopigmented mycosis fungoides is an often misdiagnosed variant characterized by hypopigmented papules and plaques without epidermal atrophy.^1^ CTCL can also trigger the autoimmune destruction of melanocytes leading to CTCL-associated vitiligo. First described in 1987,^2^ only a handful of case reports of CTCL-associated vitiligo or vitiligo-like leukoderma have been published. Amongst these, treatment success of the resultant vitiligo-like leukoderma has been variable. Herein, we present a type V skin Sézary syndrome (SS) patient with CTCL-associated vitiligo-like leukoderma who developed focal repigmentation after treatment with mogamulizumab.

## Case report

**Fig 1:**
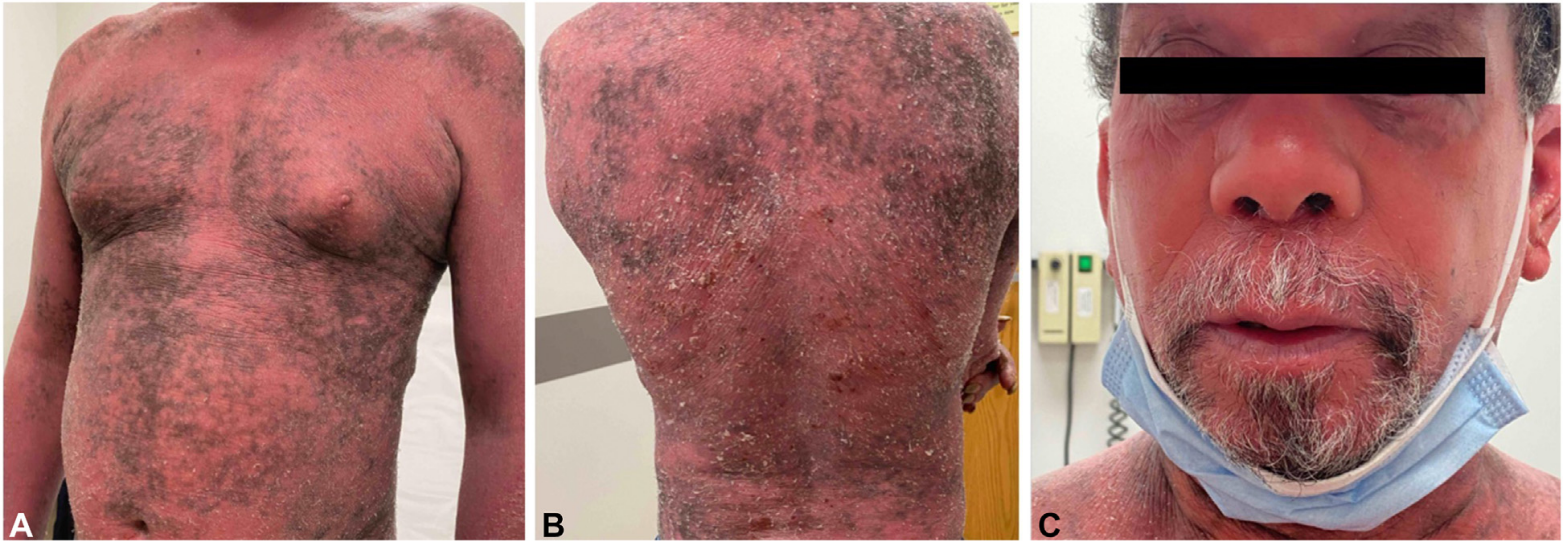
**A-C,** Diffusely thin pink-red scaly confluent plaques consistent with erythroderma on a background of patchy dark brown pigmentation.

A 62-year-old male presented for consultation of a diffuse rash that improved with 3 sessions of phototherapy but flared upon discontinuation due to transportation barriers. One year prior, he had undergone an excisional lymph node biopsy which showed small to intermediate CD4+/CD5+/CD8-/CD10- lymphocytes suggestive of a peripheral T-cell lymphoma. On examination, there were confluent deep pink scaly plaques diffusely involving greater than 95% body surface area (BSA) on a background of patchy dark brown pigmentation, significant hyperkeratosis of the bilateral plantar feet, as well as palpable cervical and supraclavicular lymphadenopathy (Fig 1). The patchy brown pigmentation involved less BSA than the erythrodermic skin; the patient’s mostly depigmented skin was noticeable because of his background type V skin. He had associated leukotrichia as well which served as a clue that his skin and hair were undergoing depigmentation. Laboratory evaluation revealed a white blood cell count of 21.3 × 10^9^/L, serum lactate dehydrogenase (LDH) of 394 IU/L, and absence of human T-lymphotropic virus HTLV-1/2 DNA. Peripheral flow cytometry demonstrated an abnormal CD4+ population (34% of all lymphocytes, absolute count 15.5 × 10^9^/L) with TRBC1 expression, a CD4:CD8 ratio of 33:1, and loss of CD7/CD8/CD26/CD56/CD57/CD16. A staging computed tomography (CT) scan showed extensive lymphadenopathy. A diagnosis of Sezary syndrome (SS) was made (TNM stage IVB, T4N3B1M0).

**Fig 2:**
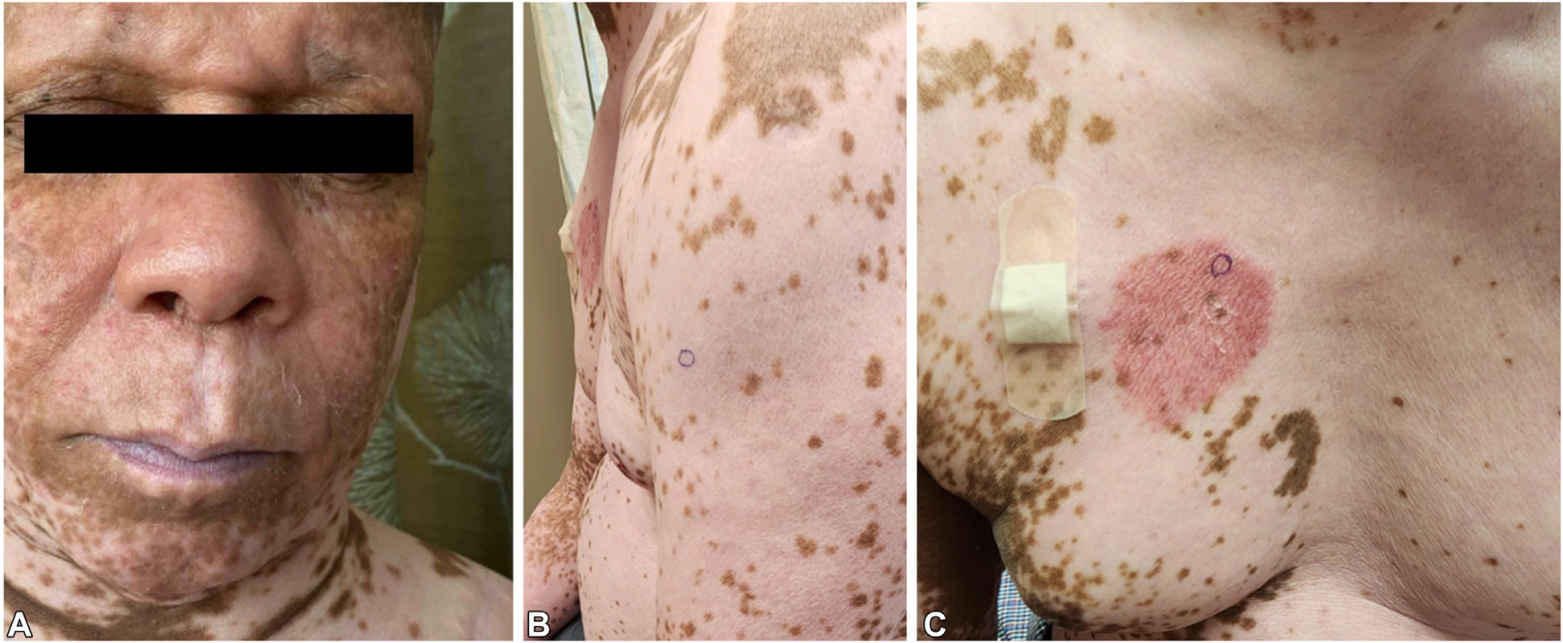
Interval improvement with (**A**) repigmentation of the face as well as biopsy sites of (**B**) a depigmented patch of the left upper arm and (**C**) a new pink scaly plaque of the right chest.

The patient was started on oral methotrexate but continued to have persistent erythroderma, LDH elevation, and a circulating Sézary cell count >2.0 × 10^9^/L. Further treatment options were considered, including bexarotene, alfa interferon, and other immunologic therapies; the patient elected to discontinue methotrexate and proceed with intravenous mogamulizumab. As the patient began to show sustained clinical improvement on mogamulizumab, it became apparent that his skin was diffusely depigmented as the erythroderma improved. Moreover, areas that were previously erythrodermic had started to repigment (Fig 2, *A*). In this setting, two 4 mm punch biopsies were performed, one of a depigmented patch (left upper arm, Fig 2, *B*) and one of a new pink plaque (right chest, Fig 2, *C*).

**Fig 3:**
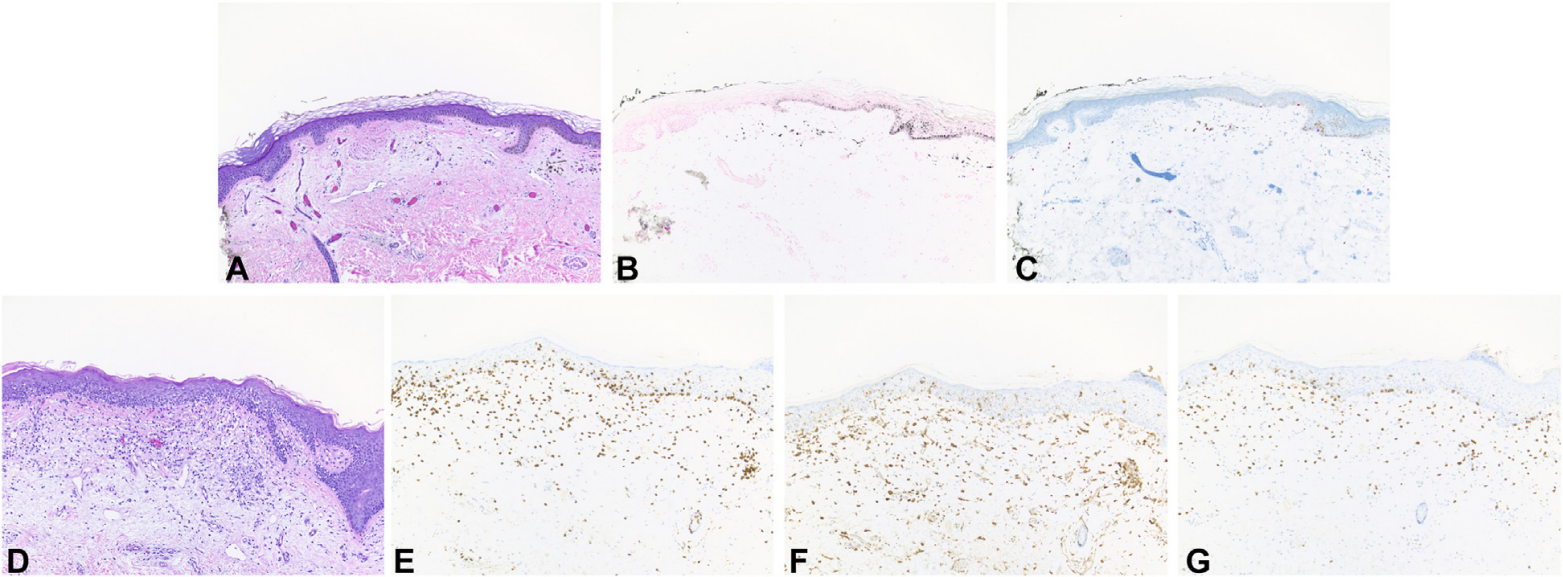
Histology section of left upper arm (vitiligo-like leukoderma) with (**A**) H&E, (**B**) Fontana Masson, and (**C**) Sox10 stain (note retention versus loss of pigment and melanocytes, respectively, on the right versus left of the slide); and of the right chest (CTCL) with (**D**) H&E, (**E**) CD3, (**F**) CD4, and (**G**) CD8 stain.

The left upper arm biopsy showed near total loss of basilar melanocytes (Fig 3, *A*-*C*). A diagnosis of SS-associated vitiligo-like leukoderma was made. The right chest wall biopsy showed spongiotic and vacuolar interface dermatitis composed of lymphocytes with mild to moderate cytologic atypia, rare eosinophils with papillary dermal fibrosis, epidermotropism, and dermal edema consistent with CTCL (Fig 3, *D*). A CD4:CD8 ratio of 5:1 was observed (Fig 3, *E*-*G*), with mildly reduced expression of CD5 and CD7, and CD30 highlighted <5% of cells overall. The patient has continued on mogamulizumab infusions with undetectable disease on peripheral flow cytometry for nearly two years with continued repigmentation and minimal topical corticosteroid use for recurrent skin disease.

## Discussion

We describe a case of a patient with type V skin and SS-associated vitiligo-like leukoderma whose diagnosis became apparent in the setting of erythroderma resolution and repigmentation while on mogamulizumab. The first reported case of CTCL-associated vitiligo was in 1987 in a 62-year-old male whose vitiligo evolved during the course of erythrodermic SS.^3^ In both this^2^ and the second ever reported case series,^4^ the authors hypothesized that destruction of melanocytes may have been induced by cell-mediated immunity related to the presence of cytotoxic T cells, possibly directly from tumoral lymphocytes or reactive T lymphocytes directed against melanocytic antigens, or indirectly from autoantibodies produced by B cells after stimulation by T-helper cells. Knol et al^3^ demonstrated through immunohistochemical studies that vitiligo in SS may be related to the presence of CD8+ T cells that are reactive against melanocyte differentiation antigens. This remains the leading hypothesis regarding the pathogenesis of vitiligo in patients with CTCL. Reported cases in the literature of vitiligo and vitiligo-like leukoderma in CTCL patients are reviewed in Table I.

**Table I:** Reported cases of vitiligo or vitiligo-like leukoderma in CTCL

Report	Age, Sex	Skin type	Clinical description	Clinical diagnosis	Treatments prior to vitiligo or vitligo-like leukoderma diagnosis	Vitiligo or vitiligo-like leukoderma latency	Treatments after vitiligo or vitiligo-like leukoderma diagnosis
Alcalay et al, 1987^2^	62M	-	Erythroderma and exfoliative dermatitis, lymphadenopathy	Sézary syndrome	Topical steroids	0.5 years	Prednisone, chlorambucil, cloxacillin
Bouloc et al, 2000^4^	83F	III	Erythroderma and generalized pruritus with poikiloderma	Sêzary syndrome	-	4.5 years	Topical and systemic steroids, topical meclorethamine, chlorambucil
	65F	II	Generalized pruritus and erythroderma, lymphadenopathy	Sézary syndrome	Topical mechlorethamine, topical and systemic steroids, cytapheresis, electron beam	5 years	Systemic steroids, chlorambucil, cytapheresis
	65M	II	Erythroderma	MF	Topical mechlorethamine, topical and systemic steroids, PUVA, chlorambucil	3 years	Methotrexate
	63M	-	Erythematous plaques, erythroderma	MF	-	10 years	Topical mechlorethamine
Macheiner et al, 2003^9^	50M	-	Erythroderma and pruritus	Sézary syndrome	Psoralen, UVA, radiation, vinblastine/cyclophosphamide, oral steroids	9 years	Extracorporeal photochemotherapy
Knol et al, 2005^3^	71F	V	Generalized erythroderma with generalized pruritus	Sézary syndrome	Interferona alpha, PUVA, oral acitretin	0.5 years	-
Zheng et al, 2018^10^	61M	-	Papules and nodules on the head, neck, and upper trunk	CD56+ cutaneous lymphoma	Pembrolizumab for melanoma prior to diagnosis of CTCL	−0.5 years	Radiation therapy, pralatrexate

*CTCL*, Cutaneous T-cell lymphoma.

Given limited reports, it may be possible that there are instances where patients are misdiagnosed by the clinical mimicker known as hypopigmented mycosis fungoides. Hypopigmented MF is distinct in that epidermotropic neoplastic cells tend to have a CD8+ phenotype, while the majority of CTCL cases, including in our patient, tend to demonstrate a CD4+ phenotype. Notably, however, the epidermal lymphocytic infiltrate in vitiligo is also composed of a CD8+ cytotoxic T cells.^5^ It is possible that there are common cytotoxic processes that lead to depigmentation in both hypopigmented mycosis fungoides and SS-associated vitiligo-like leukoderma. In this case, the change in pigmentation may be secondary to an intense interface dermatitis. Given the lack of evidence of autoimmune genesis from B cell dysfunction seen in true vitiligo, we considered if this presentation was due to cytotoxic and malignant T cells at the interface with intense inflammation, and therefore this patient was favored to have vitiligo-like leukoderma.

The repigmentation observed in our patient was noted first in the head and neck regions, but then became more noticeable elsewhere on the body, including the extremities, trunk, and genitalia, after mogamulizumab infusions, which suggests that this treatment may have treated both CTCL and CTCL-associated vitiligo-like leukoderma. This is the first case reporting this trend. Interestingly, mogamulizumab has been associated with the induction of vitiligo during treatment,^6^ but in our patient, vitiligo-like leukoderma was present prior to infusion. The mechanism of action of mogalmulizumab-induced vitiligo remains unclear, but it is possible that targeting of CCR4 which is present on regulatory T cells, which normally help maintain immune tolerance by controlling the activity of self-reactive T cells, may lead to an unintentional increase in autoimmune activity.^6^

It is important to note that patients with skin of color who have CTCL have poorer outcomes than their counterparts^7^ and this is thought to be due to under-recognition and diagnostic delay. There can be a significant impact of skin color on lesion morphology, and polymorphic presentations can make diagnosis difficult.^8^ Conversely, the presence of vitiligo-like leukoderma in this case may have been more easily appreciated against a contrasting skin tone, whereas it may remain underdiagnosed in fair skinned individuals due to a more subtle contrast.

Treatment of CTCL-associated vitiligo-like leukoderma remains poorly understood. Oftentimes, the cornerstone of therapy is addressing the underlying cutaneous lymphoma, which includes topical and systemic-directed modalities. Reports of successful repigmentation are limited. Our case demonstrates that mogamulizumab can and should be considered on the treatment ladder of patients with these concomitant diagnoses.

## Conflicts of interest

None disclosed.
